# Prehospital Systolic Hypertension and Outcomes in Patients with Spontaneous Intracerebral Hemorrhage

**DOI:** 10.7759/cureus.998

**Published:** 2017-01-26

**Authors:** Stacy Hatcher, Connie Chen, Prasanthi Govindarajan

**Affiliations:** 1 Department of Surgery, University of California San Francisco; 2 Internal Medicine, Stanford Healthcare; 3 Emergency Medicine, Stanford Healthcare

**Keywords:** stroke, emergency medical services, hypertension, ct, intracerebral hemorrhage, prehospital care, prehospital life support

## Abstract

**Background:**

It is well known that hematoma volume and expansion is associated with poor outcomes in patients with spontaneous intracerebral hemorrhage (sICH). The factors associated with hematoma volume and possible expansion include the use of anticoagulant medications, autoimmune or bacterial diseases that reduce platelet production, and genetic defects of Von Willebrand factor causing inhibition or reduction of platelet aggregation.

However, little is known about the role of elevated systolic blood pressure (SBP) on hematoma volume and its ultimate role on sICH when identified in the prehospital setting. Our objectives were to determine the prevalence of elevated SBP among diagnosed sICH patients transported by emergency medical services (EMS), and to explore possible associations between prehospital elevated SBP and hematoma volume.

**Methods:**

This is a hypothesis-generating study for which we used a retrospective observational design. The subjects included 243 adult patients who were seen and treated for sICH in an emergency department serving a county hospital in a large metropolitan city. Elevated SBP in the setting of sICH was defined as ≥140 mm Hg. A univariate analysis was performed to investigate associations between patient demographics, elevated SBP, and sICH characteristics with the pre-determined outcome of hematoma volume. We then performed a multivariable logistic regression model to determine if elevated prehospital SBP remained associated with hematoma volume.

**Results:**

The number of subjects with a hospital-based diagnosis of sICH was 243. Of those, 193 (79%) were transported by an ambulance. Among those transported by ambulance, 180 (93%) had a documented prehospital SBP; out of those patients with a documented SBP, 173 (96%) showed an elevated SBP of ≥140 mm Hg, and 82 (46%) had a hematoma volume of ≥30 mL.

Our univariate analysis showed that sICH patients with an elevated prehospital SBP of ≥140 mm Hg were associated with hematoma volume. The multivariable regression model showed that elevated prehospital SBP (≥140 mm Hg) was associated with larger hematoma volumes (odds ratio (OR) 3.86 95% confidence interval (CI) 1.02-4.60).

**Conclusions:**

Prehospital elevated SBP is associated with larger hematoma volume in patients with sICH. Future studies should confirm these findings in a larger cohort of patients.

## Introduction

Spontaneous nontraumatic intracerebral hemorrhage (sICH) is a neurovascular emergency and constitutes about 10% of all strokes [1]. It is also a leading cause of disability and mortality; approximately 40% of sICH patients die within 30 days [2]. Prognostic factors associated with hematoma volume and possible expansion such as the use of anticoagulant medications, autoimmune or bacterial diseases that reduce platelet production, and genetic defects of Von Willebrand factor causing inhibition or reduction of platelet aggregation have been studied over the years and have shown to be associated with poor outcomes [3-4]. Mortality rates, however, have not changed significantly. Control of hematoma expansion may hold the most promise as a therapeutic target. While traditional models indicate direct injury to neurons from the hematoma as a contributing factor of poor patient outcomes, the recent focus has shifted in favor of factors that might aggravate secondary brain injury such as early hematoma expansion, edema, and neurohemoinflammation.

Hematoma expansion is known to occur in the first hour after a baseline CT scan in a third of patients with sICH and is associated with significant neurodeterioration, a predictor of morbidity and mortality [5-7]. A literature review conducted by Morgenstern, et al. corroborated similar findings in which elevated SBP (>140 mm Hg) within 12 hours post sICH was associated with significantly higher mortality and morbidity [8].

Inhospital association between elevated SBP and neurodeterioration has been studied, [7,9], and analyses conducted by Qureshi, et al. have showed an association between mortality and initial mean arterial pressure (MAP), (MAP=Diastolic Blood Pressure + 1/3 Pulse Pressure), but did not show a significant association between elevated initial MAP and early neurologic deterioration [6]. Consequently, elevated prehospital SBP and its association with hematoma volume, disability and mortality are yet to be clearly determined.

The multicenter observational pilot study 'Antihypertensive Treatment of Acute Cerebral Hemorrhage-I' (ATACH I), and the 'Intensive Blood Pressure Reduction in Acute Cerebral Haemorrhage-I' (INTERACT I) pilot trial showed that early and aggressive lowering of blood pressure was feasible and reasonably safe, but ultimately did not result in any appreciable decrease in the rate of death, severe disability, or hematoma growth [10-11]. Analysis of the modified Rankin scale data collected in the INTERACT II trial did however indicate better functional outcomes when aggressive SBP lowering was instituted compared to the control group [12-14].

These trials formed the basis for the randomized Phase III clinical trial ATACH II, which sought to determine the efficacy of early and intensive blood pressure lowering treatment administered within three hours of sICH onset and continued for the next 24 hours. Similar to the ATACH I and INTERACT I and II studies, recently published results have shown no appreciable decrease in the rate of death, or disability [10-12, 15-16].

We aim to identify the prevalence of prehospital elevated SBP as defined by ≥140 mm Hg and determine if an association exists between elevated prehospital SBP and hematoma volume. This study, while exploratory in nature, may shed light on the contribution of prehospital factors in ultra-early management of sICH and its associated outcomes, as well as expand the horizon of possible factors that could contribute to improved outcomes in sICH patients.

## Materials and methods

### Study design and setting

This is a three-year retrospective observational cohort study of patients admitted through a single emergency department with a new diagnosis of sICH confirmed via computed tomography (CT) scan in the hospital. Subject enrollment and data acquisition are described previously [17-18]. All aspects of this study were approved by the Committee on Human Research of the University of California, San Francisco. Informed consent was waived because of minimal patient risk and because only deidentified data were included.

### Selection of participants

We included all ambulance-transported subjects with a final inhospital diagnosis of sICH. Interhospital transfers were excluded.

### Methods of measurement, data collection and processing

The patient demographic variables used for analysis included date of birth, gender, ethnicity, and race. The baseline variables included SBP recording and pre-sICH functional status and baseline characteristics, including modified Rankin Score (mRS) and use of anticoagulants. Post-sICH characteristics recorded include hematoma volume, location of the hemorrhage, and presence of intraventricular hemorrhage. The patient outcomes during and following hospitalization were available for analysis.

### Study definition

The hematoma volume and outcomes associated with prehospital SBP of ≥140 mm Hg were analyzed. Although data on other prehospital vital signs were available, we chose to focus solely on SBP and Glasgow Coma Scale (GCS). This selective approach was done to prevent inaccurate risk estimates resulting from the use of too many independent variables in a model with a small sample size.

### Outcome measures

The primary outcome measure was hematoma volume upon initial CT at the emergency department (ED) (Table 2). The secondary outcome measure was prehospital neurologic deterioration as defined by a ≥2 point decrease in GCS between EMS and ED evaluation [17]. Both radiological and clinical outcomes were temporally related to prehospital transport.

### Primary data analysis

The patients were initially categorized by hematoma size (≥30 mL and <30 mL) [17]. The demographics were described using frequencies and percentages for dichotomous and categorical variables. We studied the association between prehospital SBP and hematoma size using a univariate analysis. Associations between key adjustment variables such as subject demographics, baseline functional status, and sICH characteristics and outcomes were also explored (Table 1). The patient demographics and ICH characteristic variables for the regression model were chosen based on both face validity and associations observed in previous research [7-12, 14, 17-23].

In our multivariate model we sought to determine if an independent association existed between components of prehospital SBP and hematoma size at initial CT when controlling for demographic characteristics, baseline functional status of the subject, and sICH characteristics. A p-value of 0.05 was considered significant. Statistical analysis was performed using STATA Version 10.0 (Statacorp, TX, USA).

## Results

The subjects included 243 adult patients who were seen and treated for sICH in an emergency department serving a county hospital in a large metropolitan city. Out of those 243 subjects, 193 were transported by ambulance and had a mean age of 66 years (SD+/-14.5). Nintey-eight (51%) were male. A total of 180 (93%) patients had a documented prehospital SBP, out of which 173 (90%) showed an elevated SBP of ≥140 mm Hg, and 82 (43%) had a hematoma volume of ≥30 mL. The mean hematoma volume according to the CT scan on admission was 41 mL (SD=47 mL) (Table 1).

The univariate analysis (Table 2) showed that elevated prehospital SBP ≥140 mm Hg was associated with a hematoma volume of ≥30 mL. The changes reported in hematoma volume were calculated based on volume at initial CT and CT 24 hours later (OR 4.21 95% CI 1.17-15.13), presence of hematoma volumes of ≥30 mL at initial CT scan (OR 4.00 95% CI 2.18-7.28), and a negative change in functional status leading to severe disability as measured by mRS score of 4-5. Severe disability was noted at 30 days compared to an initial patient evaluation of moderate disability as measured by mRS score of three (OR 4.14 95% CI 1.52-11.57). The factors associated with higher 30-day mortality include presence of intraventricular hemorrhage (IVH) (OR 3.36 95% CI 1.80-6.27), increase in hematoma volume of ≥0.03 mL (OR 4.20 95% CI 1.17-15.13), and lower prehospital GCS scores of 4-12 (OR 3.40 95% CI 1.89-6.13) and <4 (OR 14.01 95% CI 3.2-62.17).

In the multivariable regression model, elevated prehospital SBP (≥140 mm Hg) was associated with a trend towards larger hematoma volume (OR 3.86 95% CI 1.02- 4.60). No statistically significant associations were noted between prehospital SBP and 30-day mortality (OR 0.10 95% CI 0.2-4.97), severe disability (OR 3.64 95% CI 1.01-13.06), and prehospital neurodeterioration (OR 1.22 95% CI 0.39-5.20) (Table 3).

**Table 1:** Background Characteristics Note: SD-Standard Deviation; Some analyses are based on fewer observations than the total n because of missing data. Neurodeterioration is defined as a ≥2 point decrease from EMS to ED.

Characteristics	All Subjects	Outcome at 30 days		p-value
	n=193 (%)	Alive	Deceased	
		n=97	n= 96	
Age (years)				
Mean (SD, Min-Max)	66 (14.5, 25-94)	65.4 (14.5)	67.4 (14.5)	0.03
Sex				
No. (%) Male	98 (51%)	52 (53%)	38 (49%)	0.22
Race/ Ethnicity				
White	68 (34%)	34 (34%)	35 (38%)	0.28
Black	36 (19%)	21 (23%)	15 (15%)	0.84
Asian	82 (43%)	40 (41%)	42 (44%)	0.34
Other non-white	5 (3%)	2 (2%)	3 (3%)	0.44
Pre-Rankin ≥4	9 (5%)	0 (0%)	9 (9%)	<0.01
Prehospital GCS				
Mild (13-15)	81 (44%)	58 (68%)	13 (19%)	<0.01
Moderate (4-12)	43 (23%)	14 (30%)	29 (59%)	<0.01
Severe (<4)	62 (33%)	3 (2%)	59 (23%)	<0.01
First GCS in ED				
Mild (13-15)	81 (42%)	67 (72%)	14 (14%)	<0.01
Moderate (4-12)	86 (45%)	24 (26%)	62 (62%)	<0.01
Severe (<4)	24 (13%)	2 (2%)	22 (25%)	<0.01
Neurodeterioration during EMS transport	45 (24%)	18 (20%)	27 (27%)	<0.01
Prehospital systolic blood pressure				
Mean (SD)	185 (41)	179 (4)	190 (4)	0.05
Hematoma volume (mL)				
Mean (SD)	41 (47)	17 (2)	62 (5)	<0.01
Hematoma volume < 30cm^3^	109 (57%)	68 (75%)	41 (41%)	<0.01
Hematoma volume ≥30 cm^3^	82 (43%)	23 (25%)	59 (59%)	<0.01
Intraventricular hemorrhage present	123 (64%)	46 (50%)	77 (77%)	<0.01
Infratentorial bleed	38 (20%)	18 (19%)	20 (20%)	0.68

**Table 2:** Logistic Regression Model on Hematoma Volume GCS-Glasgow Coma Scale, CI-Confidence Interval, OR-Odds Ratio, mRS-Modified Rankin Score, ICH Volume-Intracranial Hemorrhage Volume, IVH-Intraventricular Hemorrhage; Outcomes: hematoma volume (dichotomize at 30 cm^3), Prehospital neurodeterioration (decrease in GCS by ≥2 points between EMS and ED arrival), Disability (change from <4 to ≥4 on mRS), Primary predictor variable: systolic blood pressure ≥140mm Hg, Controls: age ≥80y, gender, race, pre-Rankin, location-infratentorial, volume ≥30, and presence of IVH.

	Univariate Analysis			Multivariate Analysis		
Predictor	OR	95% CI	p-value	OR	95% CI	p-value
Age >80 years	1.35	0.68-2.68	0.39	1.11	0.53-2.37	0.78
Female	1.09	0.61-1.94	0.77	1.11	0.59-2.06	0.75
Asian	1.02	0.57-1.84	0.93	0.63	0.32-1.24	0.18
Black	0.37	0.16-0.84	0.02	0.32	0.13-0.83	0.02
Other non-white	2.03	0.33-12.47	0.45	1.02	0.13-8.08	0.99
Pre-event mRS ≥4	0.66	0.16-2.69	0.55	0.54	0.10-2.82	0.46
Infratentorial location	0.07	0.02-0.27	<0.01	-	-	-
IVH present	1.80	0.97-3.34	0.06	-	-	-
Prehospital GCS (4-12)	2.00	1.11-3.62	0.02	2.22	1.11-4.46	0.03
Prehospital GCS <4	1.38	0.58-3.26	0.46	2.22	0.85-5.79	0.10
Prehospital SBP > 140 mmHg	4.21	1.17-15.13	0.03	3.86	1.02- 4.60	0.05

**Table 3:** Logistic Regression Model for Death GCS-Glasgow Coma Scale, CI-Confidence Interval, OR-Odds Ratio, mRS-Modified Rankin Score, ICH-Intracranial Hemorrhage, IVH-Intraventricular Hemorrhage; Outcome: mortality (yes/no), Primary predictor variable: systolic blood pressure ≥140mm Hg, Controls: age ≥80y, gender, race, pre-rankin, presenting GCS  ≥3, 4-12, ≥13, location-infratentorial, volume ≥30 and presence of IVH.

	Death					
	Univariate Analysis			Multivariate Analysis		
Predictor	OR	95% CI	p-value	OR	95% CI	p-value
Age >80 years	0.84	0.42-1.67	0.63	0.66	0.27- 1.62	0.36
Female	1.16	0.66-2.03	0.61	0.10	0.47- 2.13	0.10
Asian	1.16	0.66-2.03	0.62	0.90	0.39- 2.12	0.82
Black	0.67	0.32-1.39	0.29	1.09	0.35- 3.39	0.88
Other non-white	1.53	0.25-9.42	0.65	0.78	0.09- 6.53	0.82
Pre-event mRS ≥4	-	Co-linear	-	-	-	-
ICH volume > 30 mL	3.98	2.18-7.28	<0.01	4.84	1.97- 11.90	<0.01
Infratentorial location	1.01	0.50-2.06	0.97	1.61	0.49- 5.25	0.43
IVH present	3.36	1.80-6.27	<0.01	2.90	1.30- 6.49	0.01
Prehospital GCS 4-12	3.40	1.89-6.13	<0.01	6.27	2.82- 13.92	<0.01
Prehospital GCS <4	14.01	3.2-62.17	<0.01	30.86	5.86- 162.49	<0.01
Prehospital SBP > 140 mmHg	0.49	0.60-0.40	0.34	0.10	0.20- 4.97	0.10

## Discussion

A national study noted that initial SBP in patients presenting to the ED was elevated at ≥140 mm Hg in 69% of stroke patients [9]. In our sICH-only cohort we noted a higher (91%) proportion of prehospital systolic hypertension. The higher proportion may be due to our inclusion of only sICH patients versus a cohort including all stroke types.

Further studies on this topic have shown mixed results. The INTERACT I pilot showed hematoma attenuation over 72 hours in the treatment group (aggressive blood pressure (BP) lowering) within six hours of sICH onset [11]. However, the final results of the INTERACT II trial showed no significant difference in hematoma growth between treatment and control groups. Analysis of INTERACT II mRS scores did however suggest that intensive treatment improved functional outcomes [16]. A post hoc analysis by Jauch EC, et al. similarly did not show any associations between hemodynamic parameters and hematoma growth but states that this association still remains unclear and warrants further research [13].

An interesting study by Fan, et al. (2015) showed that compared to inhospital blood pressure readings, increased on-scene systolic, diastolic, and mean arterial pressures were all independently and more strongly associated with increasing neurologic deterioration as defined by ≥2 decrease in GCS within 24 hours after ED admission [19].

Differing results between other studies described could be attributed to differences in demographics of the patient population and study settings. The main limitation for this study was our small sICH cohort size as evidenced by wide confidence intervals.

ICH is seldom diagnosed in the prehospital setting due to lack of imaging modalities. CT equipped ambulances are available in parts of Europe and the United States and may play an increasing role in prehospital stroke care in the future. While we acknowledge these limitations, this is one of the few studies exploring the impact of prehospital SBP on sICH patient outcomes. Based on our findings, we hope to provide valuable information and spur investigation into possible future diagnostic and treatment modalities for sICH patients in the prehospital setting.

## Conclusions

Prehospital elevated SBP is associated with larger hematoma volume on ED arrival in patients with spontaneous intracranial hemorrhage. While these findings are limited by the size of the cohort and unmeasured variables such as prehospital hematoma volume in the early phase of care, future studies using larger sample size, accounting for further prehospital variables, and using a cumulative dose of hypertension (the area calculated under the curve of contiguous MAP values that surpass a set threshold over a certain amount of time) rather than episodic elevations in SBP are likely to shed more light on the association between prehospital SBP and sICH patient outcomes.
